# Different definitions of atopic dermatitis: impact on prevalence estimates and associated risk factors†

**DOI:** 10.1111/bjd.17853

**Published:** 2019-06-26

**Authors:** T. Nakamura, S. Haider, S. Colicino, C.S. Murray, J. Holloway, A. Simpson, P. Cullinan, A. Custovic, the STELAR investigators

**Affiliations:** ^1^ Department of Paediatrics Imperial College London London U.K.; ^2^ National Heart and Lung Institute Imperial College London London U.K.; ^3^ Division of Infection, Immunity and Respiratory Medicine University of Manchester Manchester U.K.; ^4^ Human Development and Health, Faculty of Medicine University of Southampton Southampton U.K.

## Abstract

**Background:**

There is no objective test that can unequivocally confirm the diagnosis of atopic dermatitis (AD), and no uniform clinical definition.

**Objectives:**

To investigate to what extent operational definitions of AD cause fluctuation in the prevalence estimates and the associated risk factors.

**Methods:**

We first reviewed the operational definitions of AD used in the literature. We then tested the impact of the choice of the most common definitions of ‘cases’ and ‘controls’ on AD prevalence estimates and associated risk factors (including filaggrin mutations) among children aged 5 years in two population‐based birth cohorts: the Manchester Asthma and Allergy Study (MAAS) and Asthma in Ashford. Model performance was measured by the percentage of children within an area of clinical indecision (defined as having a posterior probability of AD between 25% and 60%).

**Results:**

We identified 59 different definitions of AD across 45 reviewed studies. Of those, we chose four common ‘case’ definitions and two definitions of ‘controls’. The prevalence estimates using different case definitions ranged between 22% and 33% in MAAS, and between 12% and 22% in Ashford. The area of clinical indecision ranged from 32% to 44% in MAAS and from 9% to 29% in Ashford. Depending on the case definition used, the associations with filaggrin mutations varied, with odds ratios (95% confidence intervals) ranging from 1·8 (1·1–2·9) to 2·2 (1·3–3·7) in MAAS and 1·7 (0·8–3·7) to 2·3 (1·2–4·5) in Ashford. Associations with filaggrin mutations also differed when using the same ‘case’ definition but different definitions of ‘controls’.

**Conclusions:**

Use of different definitions of AD results in substantial differences in prevalence estimates, the performance of prediction models and association with risk factors.

**What's already known about this topic?**

- There is no objective test that can unequivocally confirm the diagnosis of atopic dermatitis (AD) and no uniform clinical definition.
- This results in different definitions utilized in AD studies, raising concerns on the generalizability of the results and comparability across different studies.

**What does this study add?**

- This study has shown that different definitions of ‘cases’ and ‘controls’ have major impacts upon prevalence estimates and associations with risk factors, including genetics, in two population‐based birth cohorts.
- These findings suggest the importance of developing a consensus on AD definitions of both ‘controls’ and ‘cases’ to minimize biases in studies.

## Abstract 2

https://www.bjdonline.com/article/different-definitions-of-atopic-dermatitis-impact-on-prevalence-estimates-and-associated-risk-factors/

**Linked Comment:** https://doi.org/10.1111/bjd.18303.

https://doi.org/10.1111/bjd.18571 available online

Although atopic dermatitis (AD) is one of the most common skin diseases,^1^ there is no universally accepted definition of this condition for epidemiological and genetic studies,^2^ and no objective test that can unequivocally confirm the diagnosis.^3^ Despite efforts to reach a consensus on nomenclature, two terms (AD and eczema) currently coexist to describe a clinically defined, pruritic, inflammatory skin condition, characterized by chronic and relapsing dermatitis in common anatomical sites.^4^ Furthermore, the two terms are often used interchangeably.^5^ Further denominations such as atopic eczema/dermatitis syndrome^6^ have also been proposed. Kantor *et al*. have shown that AD is currently the most commonly used term, but that use of the term differs between literature in different languages and scientific disciplines.^5^ However, even when the same term (e.g. AD) is used in epidemiological^7^ and genetic^8^ studies, children are assigned as ‘cases’ and ‘controls’ using a variety of different definitions.^7^, ^8^, ^9^, ^10^ This may hinder the generalizability of the results and comparisons across different studies and geographical areas,^7^, ^11^, ^12^ and may impact on estimates of the magnitude of the effects of potential risk factors and on study conclusions. Such impact has been shown in asthma, in which variation in the definition of the primary outcome had a considerable impact on the estimated prevalence and on results of prediction models.^13^

We propose that research findings may differ substantially if different definitions of AD are used. Our aim was not to tackle which definition may be the most appropriate, but to investigate the potential consequences of using different definitions on the results of AD studies. As a first step, we reviewed the definitions of AD used in the literature. We then tested the impact of the choice of the commonly used definitions of ‘cases’ and ‘controls’ on AD prevalence estimates and associated risk factors – including filaggrin (FLG) mutations^14^, ^15^ – among children aged 5 years in two U.K. birth cohorts.

## Patients and methods

### Definitions and operationalizations of atopic dermatitis

We reviewed the case definitions of AD in 26 studies included in a meta‐analysis of genome‐wide association studies^8^ and 45 studies included in a systematic review of AD persistence.^7^ More recent studies published between 2015 and 2017 were also included through a MEDLINE search, using PubMed. Studies that fulfilled the following criteria were included: (i) prospective cohort design; (ii) AD as the primary or secondary outcome; (iii) participants aged between 0 and 18 years; and (iv) published in English. We extracted the following information: (i) definition of AD and (ii) data sources used to diagnose AD (questionnaire, physical examination or medical records).

As some definitions consisted of a combination of several data sources (e.g. both questionnaires and physical examination as in ‘parent‐reported AD confirmed by physical examination’), we decomposed those data sources for each case definition (Fig. S1; see Supporting Information). Questionnaire‐based definitions were further categorized as either ‘physician‐confirmed AD’ or ‘parent‐reported AD’. As many of the questionnaire‐based definitions utilized several clinical features of AD, such as types of symptoms or treatment used, definitions were further categorized as ‘no specific features’, ‘chronic skin condition’, ‘itchy skin condition’, ‘skin condition affecting skin creases’, ‘treatment’ and ‘other’ (e.g. age of onset). The definition of ‘control’ included children who did not fulfil the case definitions, unless studies explicitly stated the definition.

### Prevalence estimates and associated risk factors using different definitions

For the analysis of the impact of different AD ‘case’ definitions, we applied four commonly used definitions of current AD identified in the literature review (Table 1) to the data from two population‐based birth cohorts: the Manchester Asthma and Allergy Study (MAAS)^16^ and the Asthma in Ashford cohort^17^ from the U.K. STELAR consortium.^18^ A detailed description of the cohorts is provided in Appendix S1 (see Supporting Information). Both studies were approved by local research ethics committees. Written informed consent was obtained from all parents. For this analysis, we used data collected at review clinics at a comparable follow‐up age of 5 years. Validated questionnaires were administered by the interviewer to collect information on parentally reported symptoms, physician‐diagnosed illnesses and medication usage. We assessed allergic sensitization by skin‐prick tests.^19^ Genotyping was performed for two FLG mutations (Appendix S1), and children with FLG loss of function were defined as those with either the nonsense mutation R501X or frameshift mutation 2282del4.^14^, ^20^

**Table 1:** Definitions of atopic dermatitis (AD) for ‘case’ applied to the data in the Manchester Asthma and Allergy Study (MAAS) and Asthma in Ashford cohorts

Definitions for ‘cases’	Question			Response to questions
	1	2	3	
1. Physician‐confirmed AD	✓	✓		Yes to 1 and 2
2. Physician‐confirmed AD *and* chronic itchy skin condition affecting skin creases	✓	✓	✓	Yes to 1, 2 and 3
3. Chronic itchy skin condition affecting skin creases		✓	✓	Yes to 2 and 3
4. Physician‐confirmed AD *or* chronic itchy skin condition affecting skin creases	✓	✓	✓	Yes to (1 and 2) or (2 and 3) or (1, 2 and 3)

Question 1 (physician‐confirmed ever AD): ‘Has a doctor ever told you that your child had eczema?’ and ‘Has a doctor ever told you that your son or daughter has eczema?’. Question 2 (current itchy skin condition): ‘Has your child had an itchy rash at any time in the last 12 months’ and ‘In the last 12 months, has your child had an itchy skin rash? (by itchy we mean scratching or rubbing the skin)’. Question 3 (current flexural rash): ‘Has this itchy rash at any time affected any of the following places: the fold of the elbows, behind the knees, in front of the ankles, under the buttocks, around the neck, ear or eyes?’ and ‘Has this skin condition at any time affected the skin creases in the past? (by skin creases we mean fronts of elbows, behind the knees, fronts of ankles’).

In the prediction modelling, we used the following set of established predictors of AD: FLG genotype, parental AD, allergic sensitization (age 5 years) and physician‐confirmed asthma (age 5 years) (for definitions, see Appendix S1).

### Statistical methods

Firstly, we compared prevalence estimates for the four different ‘case’ definitions. We then used bivariate logistic regression analysis to assess the impact of the four AD ‘case’ and the two ‘control’ definitions on associations with FLG mutations and other risk factors. Finally, we constructed prediction models using multivariable logistic regression analysis and assessed the patterns of distributions of the posterior probabilities and the performance of prediction models following the study of Van Wonderen *et al*.^13^ Performance was measured using the percentage of children whose posterior probability was in an area of clinical indecision (25–60%),^13^ assuming that a posterior probability of 25% or less predicts a low risk of the disease and a posterior probability above 60% indicates a high risk. A sensitivity analysis was also undertaken by comparing the area of clinical indecision between 25% and 50%. The analyses of prediction models were conducted in children with complete data for the included variables. We used Stata 14.2 for all analyses (StataCorp, College Station, TX, U.S.A.).

## Results

### Search for definitions of atopic dermatitis in the literature

We reviewed 45 studies (Fig. S2; see Supporting Information) and identified 59 different operational definitions of AD (summarized in Table S1; see Supporting Information). In total, 32 studies included a cumulative estimate of AD (lifetime period), 26 used current AD (defined as the presence of AD in the previous 6, 12 or 24 months) and no time period was specified in one study. Within each definition, there was further heterogeneity (e.g. within the category of physician‐confirmed AD for cumulative prevalence we found six different definitions; Table S1). After definitions that consisted of a combination of several data sources were decomposed, further heterogeneity became apparent (31 were derived from a single data source, 24 from two, and four from three or more). Of these, 41 definitions were based on physician‐confirmed AD, 43 on parent‐reported AD, seven on physical examination and two on data from medical records.

Of the 59 operational definitions, 27 were derived based on questions referring to an ‘itchy skin condition’, 23 on ‘skin condition affecting skin creases’ and 17 on ‘chronic skin condition’. Of the 43 case definitions that included ‘parent‐reported AD’, 27 (63%) incorporated at least one of these three common features. Of these, 11 adopted all three features (Fig. S3; see Supporting Information). Of 41 definitions that included ‘physician‐confirmed AD’, 33 relied on a single or several questions pertaining to physician diagnosis (Fig. S3). Only seven definitions incorporated the use of treatment, and the age of onset was considered in four.

We then chose four common operational case definitions (Table 1) to estimate the prevalence, risk factors and predictive performance of prediction models in the two cohorts, as follows. Definition 1: physician‐confirmed AD. Definition 2: physician‐confirmed AD and parent‐reported chronic itchy skin condition affecting skin creases. Definition 3: parent‐reported chronic itchy skin condition affecting skin creases. Definition 4: physician‐confirmed AD or parent‐reported chronic itchy skin condition affecting skin creases. For these analyses, ‘controls’ were defined as children who did not fulfil the case definition.

### Prevalence estimates, associates and prediction model performance

We used data from 1069 children in MAAS and 604 in Ashford, of whom 771 (MAAS) and 405 (Ashford) had a complete dataset. Table 2 shows the characteristics of the children included in the analysis. White children accounted for 95% of the sample in MAAS and 99% in Ashford. FLG mutations were present in one‐tenth of the children.

**Table 2:** Characteristics of the study populations

Variable	MAAS	Asthma in Ashford
	*n*/*N* (%)	*n*/*N* (%)
Sex (male)	581/1069 (54)	259/499 (52)
Parent history of AD	265/1068 (25)	174/593 (29)
Paternal history of AD	112/1068 (10)	82/593 (14)
Maternal history of AD	175/1069 (16)	110/596 (18)
Dog ownership at recruitment	174/1047 (17)	155/596 (26)
Cat ownership at recruitment	219/1047 (21)	223/596 (37)
Physician‐confirmed ever AD	421/1058 (40)	214/604 (35)
Current itchy skin condition	344/1069 (32)	165/604 (27)
Current flexural rash	292/1069 (27)	89/604 (15)
Physician‐confirmed asthma	248/1062 (23)	118/604 (20)
Atopic sensitization	291/954 (30)	78/551 (14)
Ethnicity (white)	971/1023 (95)	568/574 (99)
Filaggrin null mutations	73/795 (9)	45/439 (10)

The denominators indicate children without a missing value for each variable. AD, atopic dermatitis; MAAS, Manchester Asthma and Allergy Study.

**Figure 1:**
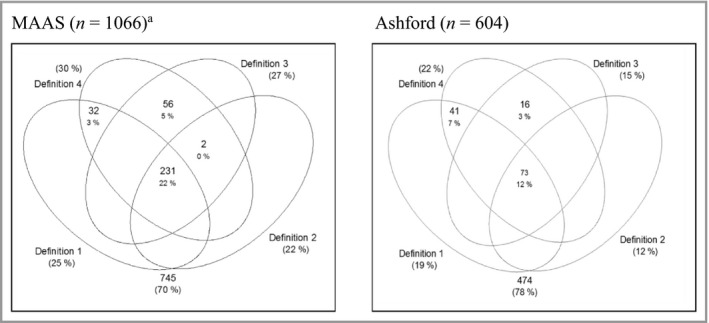
Overlap of each definition for current atopic dermatitis (AD) in the Manchester Asthma and Allergy Study (MAAS) and Asthma in Ashford cohorts. Definition 1: physician‐confirmed AD. Definition 2: physician‐confirmed AD and parent‐reported chronic itchy skin condition affecting skin creases. Definition 3: parent‐reported chronic itchy skin condition affecting skin creases. Definition 4: physician‐confirmed AD or parent‐reported chronic itchy skin condition affecting skin creases. ^a^Three children had missing values in definitions 1 and 2.

The Venn diagrams in Figure 1 show that the prevalences were highest using definition 4 [30%, 95% confidence interval (CI) 27–33 and 22%, 95% CI 18–25] and lowest using definition 2 (22%, 95% CI 19–24 and 12%, 95% CI 9–15) in MAAS and Ashford, respectively. The mean differences (95% CI) were 8% (5–12), *P* < 0·001 in MAAS and 10% (5–13), *P* < 0·001 in Ashford. The prevalence estimates (95% CI) of AD were similar using definitions 1 and 3: 25% (22–27) and 27% (25–30), respectively, in MAAS and 19% (15–22) and 15% (12–17) in Ashford. The transitions between cases and controls in each definition are shown in Table S2 (see Supporting Information). For example, among children assigned as cases in definition 4, 27% (MAAS) and 43% (Ashford) were assigned as controls in definition 2. Among those assigned as cases in definition 1, 12% (MAAS) and 36% (Ashford) were assigned as controls in definition 3.

The strength of the association with FLG genotype among white children differed between different definitions in both cohorts. The odds ratios (ORs) (95% CI) ranged from 1·8 (1·1–2·9) to 2·2 (1·3–3·7) in MAAS and from 1·7 (0·8–3·7) to 2·3 (1·2–4·5) in Ashford (Table 3). Associations with other risk factors are shown in Table S3 (see Supporting Information).

**Table 3:** Associations between filaggrin null mutations and four ‘case’ definitions of atopic dermatitis (AD) among the children of white European origin

	MAAS		Asthma in Ashford	
	OR (95% CI)	*P*‐value	OR (95% CI)	*P*‐value
Definition 1	1·9 (1·1–3·2)	0·02	2·3 (1·2–4·5)	0·02
Definition 2	2·2 (1·3–3·7)	0·003	2·2 (1·0–4·7)	0·045
Definition 3	1·8 (1·1–2·9)	0·027	1·7 (0·8–3·7)	0·15
Definition 4	1·8 (1·1–2·9)	0·02	1·9 (0·9–3·8)	0·052

Definition 1: physician‐confirmed AD· Definition 2: physician‐confirmed AD and parent‐reported chronic itchy skin condition affecting skin creases· Definition 3: parent‐reported chronic itchy skin condition affecting skin creases· Definition 4: physician‐confirmed AD or parent‐reported chronic itchy skin condition affecting skin creases· MAAS, Manchester Asthma and Allergy Study; OR, odds ratio; CI, confidence interval. OR and CI determined by binary logistic regression.

### Performance of prediction models

**Figure 2:**
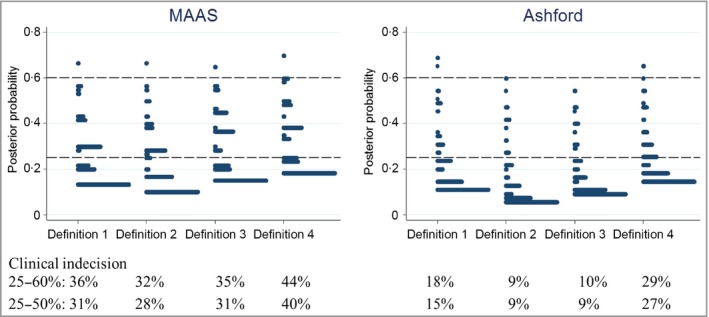
Performance of prediction models for four different ‘case’ definitions of atopic dermatitis (AD) in the Manchester Asthma and Allergy Study (MAAS) and Asthma in Ashford cohorts. Definition 1: physician‐confirmed AD. Definition 2: physician‐confirmed AD and parent‐reported chronic itchy skin condition affecting skin creases. Definition 3: parent‐reported chronic itchy skin condition affecting skin creases. Definition 4: physician‐confirmed AD or parent‐reported chronic itchy skin condition affecting skin creases. Multivariate logistic regression analysis included filaggrin mutations, parental history of AD, allergic sensitization at age 5 years and physician‐confirmed asthma at age 5 years as predictors. The area of clinical indecision represents the percentage of children whose posterior probability lies between 25% and 60% or 25% and 50%.

Figure 2 shows the distributions of posterior probabilities of the prediction models of current AD for the four definitions of ‘cases’. In both cohorts, the distribution of the probabilities varied depending on the definition. A consistent finding was that the posterior probabilities in definition 2 were skewed to the lowest, and those in definition 4 were skewed to the highest. The percentages of children whose posterior probability was in the area of clinical indecision were lowest in definition 2 (32% in MAAS and 9% in Ashford) and highest in definition 4 (44% and 29%). Hence, in both cohorts, the prediction models had the best performance in definition 2 and the worst performance in definition 4.

### The effect of different definitions of ‘controls’

We then proceeded to ascertain the effect of different definitions of ‘controls’ on the pattern of the association with risk factors. From the literature search, we extracted two definitions of ‘control’, which comprised the combination of responses to several questions (‘strict’ and ‘moderate’, Table S4; see Supporting Information). Using the ‘strict’ control definition, 186 (18%) children in MAAS and 135 (22%) in Ashford were unclassifiable (i.e. could not be assigned to either ‘case’ or ‘control’ due to a positive answer to one of the questions we used). The patterns of responses to the three questions among ‘unclassifiable’ children are shown in Table S5 (see Supporting Information).

The associations of AD (using definition 4) with FLG mutations were stronger when we used the ‘strict’ control definition (OR 2·4, 95% CI 1·5–4·0 and OR 2·2, 95% CI 1·1–4·6) than the ‘moderate’ (OR 1·8, 95% CI 1·1–2·9 and OR 1·9, 95% CI 0·99–3·8) in MAAS and Ashford, respectively (Table 4). We observed a significant association between the ‘unclassifiable’ group with FLG mutations, which was of similar magnitude to that for the cases in MAAS (OR 2·5, 95% CI 1·3–4·7) but not in Ashford (OR 1·4, 95% CI 0·7–3·2) (Table S6; see Supporting Information). The associations with other risk factors are shown in Table S7 (see Supporting Information). In both cohorts, associations with sensitization and asthma were stronger when we used the ‘strict’ control definition.

**Table 4:** Odds ratios (ORs) for the association between atopic dermatitis (AD) and filaggrin mutations in two different ‘control’ definitions using the same case definition among the children of white European origin (definition 4: physician‐confirmed AD or parent‐reported chronic itchy skin condition affecting skin creases)

Control definition	MAAS			Asthma in Ashford		
	*n* (%)	OR (95% CI)	*P*‐value	*n* (%)	OR (95% CI)	*P*‐value
Strict						
Controls	519 (64)	Reference		315 (72)	Reference	
Cases	286 (36)	2·4 (1·5–4·0)	0·001	123 (28)	2·2 (1·1–4·6)	0·03
Moderate						
Controls	685 (71)	Reference		445 (78)	Reference	
Cases	286 (29)	1·8 (1·1–2·9)	0·02	123 (22)	1·9 (0·99–3·8)	0·052

MAAS, Manchester Asthma and Allergy Study; CI, confidence interval. OR and CI determined by binary logistic regression.

As the choice of control definition may have implications for sample size and power, we calculated the power for detecting an association between AD and FLG genotype using the strict and moderate control definitions in MAAS. Although the sample size was reduced by approximately one‐fifth when moving from the moderate to strict definition, the power increased by around 50%, from 0·58 to 0·85, by having a ‘purer’ control as a comparator for cases of AD. Consequently, there was a larger effect size using the strict version compared with the moderate one (Table 4).

## Discussion

We have described numerous different definitions of AD that have been used in epidemiological and genetic studies. By applying common definitions to two population‐based birth cohorts, a consistent finding was that the use of different definitions of both cases and controls resulted in substantial differences in the prevalence estimates, the performance of prediction models and the associations with risk factors.

One limitation of this study is that our literature review was not systematic, hence relevant studies may have been missed. However, we reviewed studies encompassed within recent meta‐analyses of AD persistence^7^ and genome‐wide association studies,^8^ and our results may contribute to a discussion about the extent to which the variability in the results of these studies arose from differences in the definition of primary outcome.

We assessed the impact of questionnaire‐based definitions using three questions regarding AD features, but the questions were not identical in the two cohorts. This may account for some of the differences in findings between our cohorts. We acknowledge that physical examination may offer a more accurate way of defining AD.^7^ The U.K. Working Party^21^, ^22^, ^23^ and Hanifin and Rajka^24^ diagnostic criteria are excellent for case definition in case–patient studies, but are difficult to implement fully in large‐scale epidemiological studies, which are mostly questionnaire based. Information from physical examination available in birth cohorts is usually available at only a few time points during the clinical follow‐up (e.g. once every 2–3 years). Given the temporal variability of AD symptoms, using this information would likely introduce bias towards more severe disease. However, it is of note that in any of the data sources there are currently no uniform definitions,^25^, ^26^ and the variation of outcomes in observational studies of AD may well be more extensive than the findings reported in this study.

A further limitation is that we assessed children at age 5 years, and cannot infer that different definitions have a similar impact in other age groups. A study that investigated the association between AD and cardiovascular disease in adults reported a poor agreement between questionnaire‐based diagnostic criteria, thus hindering consistent conclusions about associations.^27^

We have not taken into account the temporal pattern of AD during childhood. Identification of the individual trajectories over the life course may contribute to understanding the disease heterogeneity,^28^ and latent class analysis has recently been used to assign children to different AD phenotypes based on longitudinal patterns of flexural rash.^29^, ^30^ It would be important to know how the different disease definitions impact on the identification of AD trajectories, but such analyses were beyond the scope of the current study.

We did not include all identified FLG mutations. However, we have previously shown in MAAS that there were no differences in results when FLG loss of function was defined using R501X and 2282del4, compared with using six mutations (R501X, S3247X, R2447X, 2282del4, 3673delC and 3702delG).^31^

When comparing the results of different cohorts, it is necessary to consider the study regions^32^ and languages^33^ and the age of the patients^34^, ^35^, ^36^ as confounders affecting the prevalence of AD. We carried out our analyses in two birth cohorts from the same geographical area, which used similar questionnaires administrated at the same age. As a result, we anticipate the effect of these confounders to be minimal.

We confirmed a wide variety of definitions for AD in the literature. The most commonly used definition was questionnaire‐reported physician‐confirmed AD (our definition 1). The second most common definition used three important features of AD, namely ‘itchy skin condition’, ‘skin condition affecting skin creases’ and ‘chronic skin condition’ (our definition 3), which may be influenced by the International Study of Asthma and Allergies in Childhood (ISAAC) core questionnaire.^37^ The ISAAC questionnaire was established in 1995 to enhance the comparability of epidemiological research in asthma and allergic diseases.^37^ However, our findings demonstrate that although many studies adopted the ISAAC questionnaire, a variety of definitions have been used (e.g. using questions on chronic itchy skin condition, but not the distribution affecting skin creases).^38^, ^39^ Williams *et al*. cautioned that such modifications may result in a decrease in the specificity of the diagnosis.^22^

FLG mutations are one of the most robust genetic risk factors for AD,^15^, ^40^ but a number of factors can mediate this relationship, including race and age.^34^ The heterogeneous patterns of associations with FLG mutations in our study populations, which are ethnically homogeneous and were assessed at the same age, indicate that different case–control definitions may have an adverse impact on our understanding of the underlying pathophysiological mechanisms. We observed in both cohorts that some definitions (such as definition 2) had stronger associations with FLG mutations than others. This definition included both physician‐confirmed AD *and* parent‐reported chronic itchy skin condition affecting skin creases. In addition, the prediction models had the best performance for definition 2, with the lowest percentage of the area of clinical indecision. An implication of this is that a standardized definition of AD should capture multiple domains of the disease, including severity.

Furthermore, the comparison between the ‘strict’ and ‘moderate’ control definitions demonstrated that the association of AD with FLG mutations was stronger if the ‘strict’ definition was used. When we used the ‘strict’ definition, one‐fifth of children were unclassifiable (and thus eliminated from the analyses). However, despite this reduction in sample size, the power of the study to detect significant associations increased by around 50%, and with a larger effect size. It is of note that even though the choice of definition of ‘controls’ for the analyses of genetic and environmental risk factors clearly influenced the study outcomes, in practice, of 28 studies utilizing multiple case definitions, only seven (25%) reported the definitions for the ‘controls’ expressly.

Given a significant association of the ‘unclassifiable’ group with FLG loss‐of‐function mutations, some of these children are likely to have mild AD, or other conditions such as ichthyosis. Some participants with FLG null mutations have fallen in the ‘unclassifiable’ group because, even though they were asymptomatic at age 5 years, a doctor had diagnosed AD in their infancy. This is consistent with a finding that the average duration of AD persistence in individuals with FLG mutation was 77 months.^41^

Our findings suggest that large questionnaire‐based studies, in which the primary outcome is usually defined using the lowest common denominator, may not be the most informative, and that it may be time to move on to clinical diagnosis. The international Harmonising Outcome Measures for Eczema initiative suggested the use of a minimum standard of core features, such as clinical signs, symptoms, long‐term control and quality of life, for clinical trials,^42^ and a similar approach is needed for epidemiological and genetic studies.

In conclusion, there is a pressing need to develop a uniform definition for ‘cases’ and ‘controls’ of AD for epidemiology using a set of harmonized outcomes. These should comprise multidimensional information to facilitate comparison of study findings and better understanding of AD heterogeneity, and to minimize biases arising from the choice of definitions.

## Supporting information


**Appendix S1.** Supplementary methods.
**Appendix S2.** Supplementary references.
**Fig S1.** Categorization of definitions in the reviewed studies.
**Fig S2.** Study selection.
**Fig S3.** Features incorporated in definitions of atopic dermatitis (AD) based on physician‐confirmed and parent‐reported AD.
**Table S1** Definitions used in the literature reviews.
**Table S2** Transition between case and control in each definition.
**Table S3** Associates of atopic dermatitis using four different ‘case’ definitions.
**Table S4** Atopic dermatitis definitions for ‘controls’.
**Table S5** Questions with a positive response among unclassifiable children in the ‘strict’ definition for ‘controls’.
**Table S6** Association of children defined as ‘unclassifiable’ in the ‘strict’ control definition with filaggrin null mutations.
**Table S7** Odds ratios for the association between atopic dermatitis and different risk factors in two different ‘control’ definitions using the same case definition.
