# Supplementary material for: Different definitions of atopic dermatitis: impact on prevalence estimates and associated risk factors
Source: Br J Dermatol. 2019 Jun 26;181(6):1272–9. doi: 10.1111/bjd.17853 (PMC6916614; doi:10.1111/bjd.17853)
Supplement: Supplementary file 1 — Appendix S1. Supplementary methods. Appendix S2. Supplementary references. Fig S1. Categorization of definitions in the reviewed studies. Fig S2. Study selection. Fig S3. Features incorporated in definitions of atopic dermatitis (AD) based on physician‐confirmed and parent‐reported AD. Table S1 Definitions used in the literature reviews. Table S2 Transition between case and control in each definition. Table S3 Associates of atopic dermatitis using four different ‘case’ definitions. Table S4 Atopic dermatitis definitions for ‘controls’. Table S5 Questions with a positive response among unclassifiable children in the ‘strict’ definition for ‘controls’. Table S6 Association of children defined as ‘unclassifiable’ in the ‘strict’ control definition with filaggrin null mutations. Table S7 Odds ratios for the association between atopic dermatitis and different risk factors in two different ‘control’ definitions using the same case definition. [file BJD-181-1272-s001.docx]

***Different definitions of atopic dermatitis: Impact on prevalence estimates and associated risk factors***

Running head: Impact of Different definitions of Atopic dermatitis

T. Nakamura*^1^, S. Haider*^1^, S.Colicino^2^, C.S. Murray^3^, J.Holloway^4^, A. Simpson^3#^, P. Cullinan ^2#^, A. Custovic^1#^

on behalf of STELAR^5^ investigators

*Equal contribution

^#^Joint senior authors

^1^Department of Paediatrics, Imperial College London, UK

^2^National Heart and Lung Institute, Imperial College, London, UK

^3^Division of Infection, Immunity & Respiratory Medicine, University of Manchester, Manchester

^4^ Human Development and Health, Faculty of Medicine, University of Southampton, Southampton

**Supplementary material**

**METHODS**

**Manchester Asthma and Allergy Study**

**Screening and recruitment:** All pregnant women were screened for eligibility at antenatal visits between 1995 and 1997. Of the 1499 couples met the inclusion criteria, 288 declined to take part and 27 were lost to follow-up between recruitment and birth of a child. A total of 1184 participants had some evaluable data.

**Follow-up:** Children have been followed prospectively, and attended review clinics at age of 1, 3, 5, 8, 11, and 16 years. Interviewer-administrated questionnaire was obtained at each follow-up. Home visits for study participants were carried out when participants were unable to attend clinic appointments.

**Definition of explanatory variables in prediction models**

*Parental history of AD:* A positive response to the question “Has a doctor ever told you that you had eczema”. The question was asked to both parents.

*Physician-confirmed asthma:* A positive response to the question “Has a doctor ever told you that your child had asthma?”

*Allergic sensitization*: At least one positive skin prick tests to house dust mite, cat, dog, pollens, molds, egg, and milk. A positive response to SPT was defined as mean weal diameter 3mm greater than negative control.

**ASHFORD**

**Screening and recruitment:** Over 18 months and starting in late 1993, every woman who presented for antenatal care to three general practices in Ashford were approached. Of 710 invited to take part in the study, 658 agreed and subsequently 625 women gave birth to 642 babies.

**Follow-up:** Children have been followed prospectively, and attended review clinic at ages 1, 2, 4, 5, and 14 years. Interviewer-administrated questionnaire was obtained at each follow-up.

**Definition of explanatory variables in prediction models**

*Parental history of AD:* A positive response to either question “Do you have or have you ever been told you have eczema?” or “The father has had or has ever been told to have eczema?”

*Physician-confirmed asthma:* A positive response to the question “Has a doctor ever told you that your son or daughter has asthma?”

*Allergic sensitization:* At least one positive response of skin prick tests to house dust mite, cat, and pollens. A positive response to SPT was defined as mean weal diameter 3mm greater than negative control.

**Definition of AD cases and controls in MAAS and Ashford**

The definitions of primary outcome were made according to responses to the following questions which were selected based on the availability and the similarity in both cohorts:

*Physician-confirmed ever AD:* “Has a doctor ever told you that your child had eczema?”; (Q1).

*Current itchy skin rash:* “Has your child had an itchy rash at any time in the last 12 months” (MAAS) and “In the last twelve months, has your child had an itchy skin rash? (by itchy we mean scratching or rubbing the skin)” (Ashford); (Q2).

*Current flexural rash:* “Has this itchy rash at any time affected any of the following places: the fold of the elbows, behind the knees; in front of the ankles, under the buttocks; around the neck, ear or eyes?” (MAAS) and “Has this skin condition at any time affected the skin creases in the past? (by skin creases we mean fronts of elbows, behind the knees, fronts of ankles” (Ashford); (Q3).

Children with positive responses to Q1 (physician-confirmed ever AD) and Q2 (Current itchy skin rash) were assigned as the case in Definition 1.

**Definition of controls**

We then proceeded to ascertain the effect of different definitions of “control” on the patterns of the association with different risk factors. From the literature search, we extracted two definitions of “control” which were comprised of the combination of responses to several questions. The “strict” definition contained negative responses to all three questions(1-3) and the “moderate” version contained at least two negative responses to the three questions(4).

Children with one positive and two negative responses to the included questions were defined as “unclassifiable” in the “strict” definition.

**Filaggrin genotyping**

Genotyping for R501X was performed using a TAQMAN-based allelic discrimination assay (Applied Biosystems). Allelic discrimination was assessed using Applied Biosystems 7700 sequence detection system. Probes and primers were as described. Mutation 2282del4 was genotyped by sizing of a fluorescent-labelled PCR fragment on an Applied Biosystems 3100 or 3730 DNA sequencer (5).

*Filaggrin null mutations:* Children carrying the minor allele for at least one of the following FLG null variants: R501X and 2282del4.

**Figure E1.** Categorisation of definitions in reviewed studies.

**
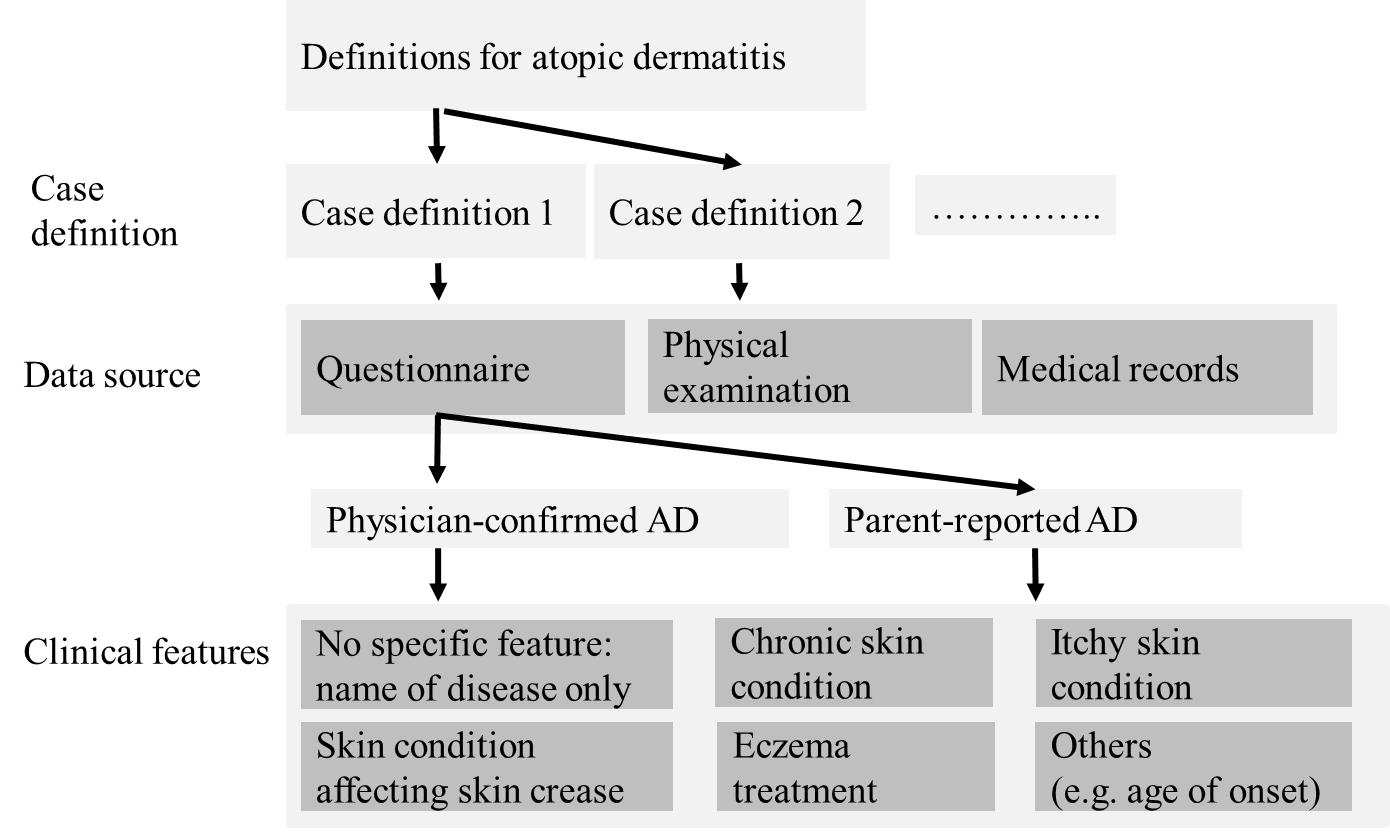
**

**Figure E2. Study selection**

**
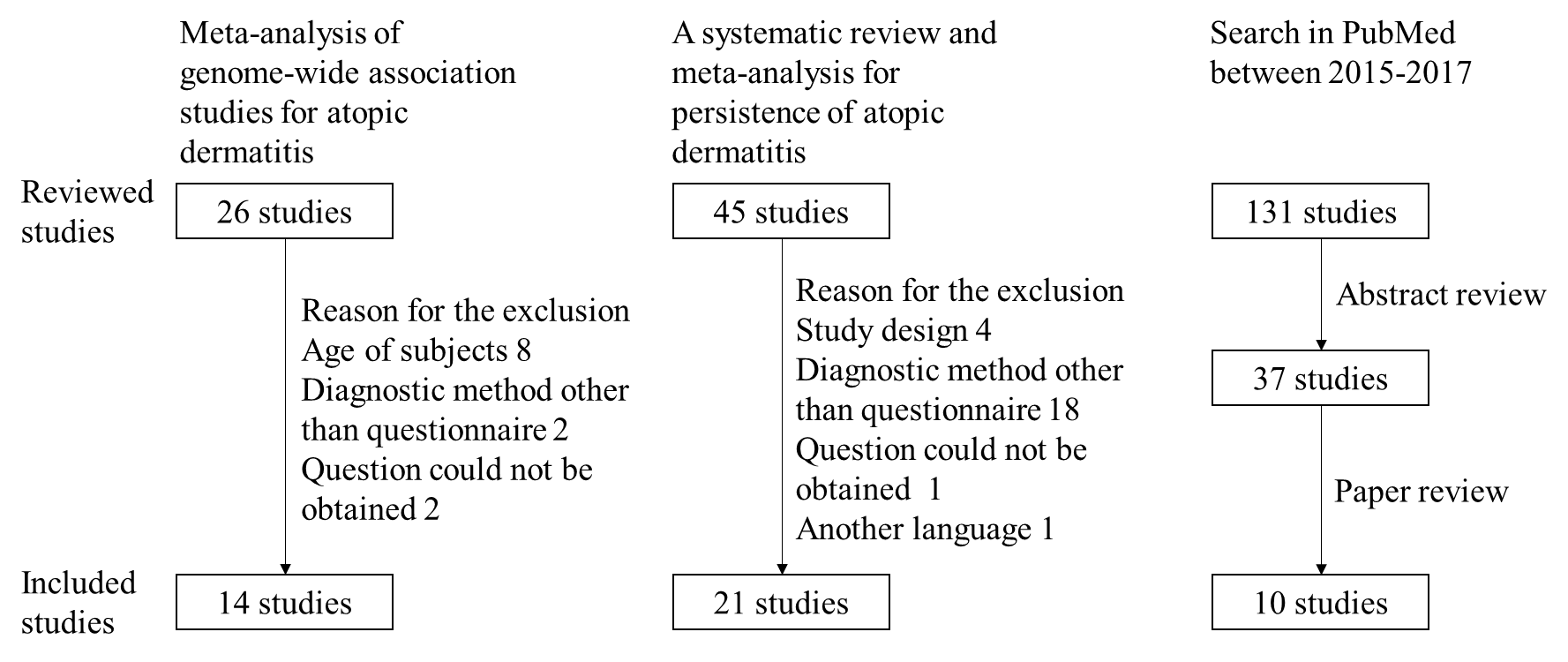
**

Meta-analysis of genome-wide association studies for atopic dermatitis was published in 2011 and included 26 studies for its discovery and replication analysis(1).

A systematic review and meta-analysis for persistent of atopic dermatitis included 45 studies extracted from several medical databases from at most 1887 to 2015(6).

Search in PubMed was conducted using the below strategy: ("Eczema"[Mesh] OR "Dermatitis, Atopic"[Mesh]) AND "Cohort Studies"[Mesh] NOT "Review"[Publication Type] AND ("2015/07/17"[PDat] : "2017/07/17"[PDat] AND "humans"[MeSH Terms] AND English[lang] AND ("infant"[MeSH Terms] OR "child"[MeSH Terms] OR "adolescent"[MeSH Terms]))

**Figure E3.** Features incorporated in AD definitions based on in physician-confirmed and parent-reported AD.


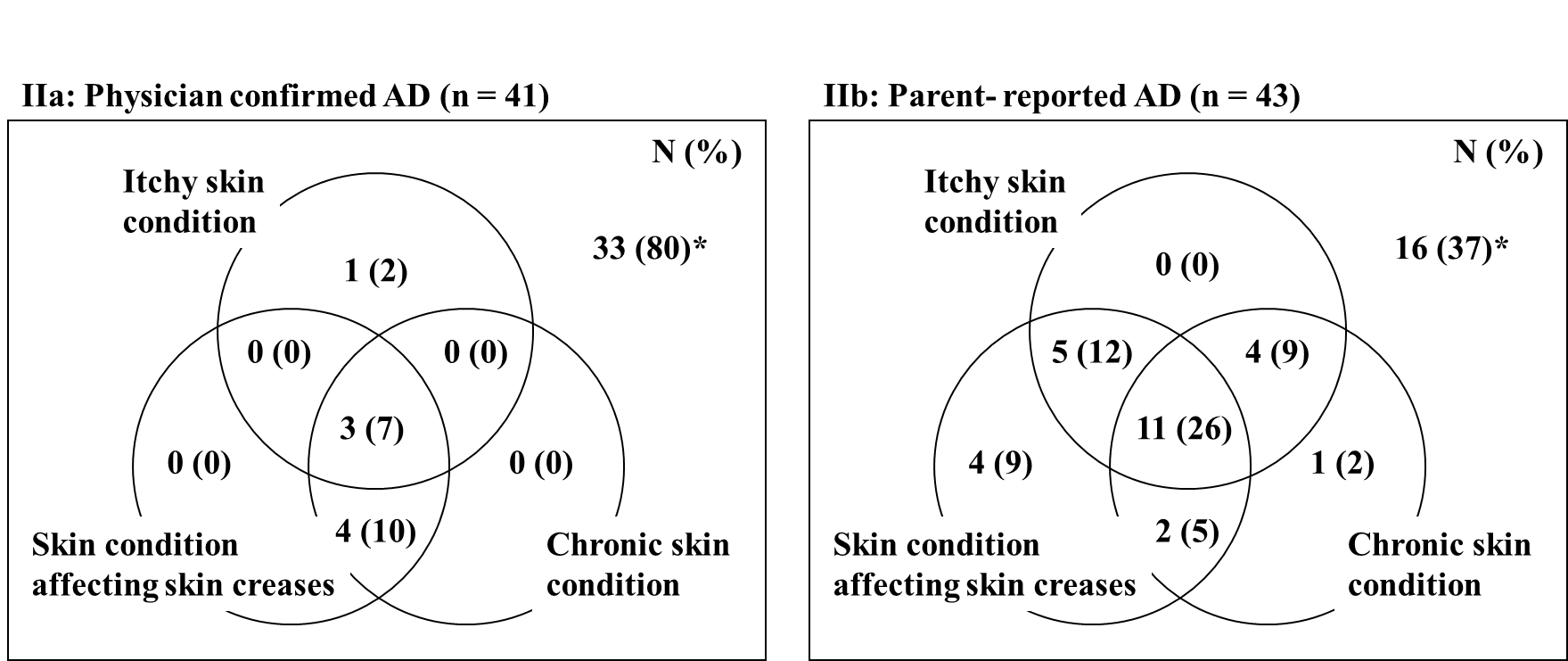


* Number and percentage of definition without questions regarding chronic skin condition, itchy skin condition, and skin condition affecting skin crease.

**Table E1. The list of definitions in the literature reviews**

**AD definitions for cumulative prevalence based on physician-confirmed AD**

| No. | Definition of eczema | Case response | Control response | Ref |
| --- | --- | --- | --- | --- |
|  | ***Physician-confirmed AD*** |  |  |  |
| 1 | 1. Has your child ever had eczema confirmed by a doctor? | Yes to 1 | No to 1 | (1, 7) |
| 2 | 1. Does your child have or has he/she had any of the following health problems? (Atopic eczema listed as item)  2. If yes, has the mother and child health centre or someone else referred your child for further specialist investigation? | Yes to 1 and 2 | No to 1 and 2 | (1, 8) |
| 3 | 1. Have you ever had eczema which has been called infantile eczema, atopic eczema or allergic eczema?  2. Have you ever been treated by a doctor?" | Yes to 1 and 2 | No to 1 | (1, 9) |
| 4 | 1. Did a physician diagnose any of the following diseases during the 1st/2nd year of life: allergic or atopic eczema/dermatitis? | Yes to 1 | No to 1 | (10) |
| 5 | 1. ever doctor-diagnosed eczema | Yes to 1 | No to 1 | (10) |
| 6 | 1. Do you think you think your child has ever had atopic dermatitis? Has anyone ever told you your child has atopic dermatitis? (Yes and who (paediatrician, GP, child health nurse, naturopath, friend, relative)  2. Has your child had atopic dermatitis in the last 12 months? | Yes to 1 and paediatrician or GP | No to 1 and 2. subjects who answered yes to 1 an were diagnosed to someone other than a paediatrician/GP were coded as missing | (1, 11) |
| 7 | 1. Has your child in the past 12 months had eczema?  2. Has a doctor ever actually said that your child has eczema? | 1. Yes, and saw Dr  2. Yes  Yes to 1 or 2 | No to 1 and 2 | (1, 12) |
| 8 | 1. Has a doctor diagnosed your child as having atopic dermatitis after age of x year  2. Has a doctor ever diagnosed your child as having atopic dermatitis up to 1 year of age | Yes to 1 or 2 | No to 1 and 2 | (1, 13) |
| 9 | 1. Did a physician since birth ever diagnosed your children with eczema?  2. Did a physician since birth ever diagnosed your children with baby eczema? | Yes to 1 or 2 | No to 1 and 2 | (1, 14) |
|  | ***Physician-confirmed AD or physical examination*** |  |  |  |
| 10 | 1. Parent report of doctor-diagnosed eczema within the first year of life  2. Eczema observed on the face, back, or elbow flexures by a trained nurse at the time of recruitment. | Yes to 1 or 2 | No to 1 and 2 | (15) |
| 11 | 1. Ever having had eczema (self-diagnosed or diagnosed by someone other than a physician)  2. Ever having been diagnosed with eczema by a physician"  3. Those have itchy rashes in skinfolds." | Yes to [1 or 2] or [1 or 3] | No to [1 and 2] or No to 3 | (16) |
|  | ***Physician-confirmed AD or use of anti-inflammatory medication and parent-reported itchy skin condition affecting skin creases or physical examination*** | | | |
| 12 | 1. The presence of a persistent for at least 2 weeks and/or chronically relapsing dry itchy skin lesion in any body area except that of the diaper, in conjunction with a relevant diagnosis (including prescription of medication) established by a physician.  2. The diagnosis was confirmed by a paediatric allergy specialist according to standard criteria. | Yes to 1 or 2 | No to 1 and 2 | (17) |

**AD definitions for cumulative prevalence based on both of physician-confirmed and parent-reported AD**

| No. | Definition of eczema | Case response | Control response | Ref |
| --- | --- | --- | --- | --- |
|  | ***Physician-confirmed AD or parent-reported chronic itchy skin condition affecting skin creases*** |  |  |  |
| 13 | 1. Itchy rash for at least 2 weeks with typical distribution and dry skin during the last year at age 1, 2, or 4 years  2. Doctor’s diagnosis of eczema sometime during the first 4 years." | Yes to 1 or 2 | No to 1 and 2 | (18) |
| 14 | 1. Itchy rash for at least 2 weeks with typical distribution and dry skin during the last year?  2. Doctor’s diagnosis of eczema from the age of 4 years? | Yes to 1 or 2 | No to 1 and 2 | (18) |
| 15 | 1. Dry skin, itchy rashes with age-specific location for 2 weeks or more the year before follow-up  2. Doctor’s diagnosis of eczema since the last follow-up." | Yes to 1 or 2 | No to 1 and 2 | (19, 20) |
|  | ***Physician-confirmed AD or parent-reported itchy skin condition affecting skin creases*** |  |  |  |
| 16 | 1. Dry skin in combination with itchy rash and typical localization the year before follow-up?  2. Doctor’s diagnosis of eczema since the X follow-up | Yes to 1 or 2 | No to 1 and 2 | (19, 20) |
| 17 | 1. Dry skin in combination with itchy rash and typical localization the year before follow-up  2. Doctor’s diagnosis of eczema since the age of 10 years." | Yes to 1 or 2 | No to 1 and 2 | (19, 20) |
|  | ***Physician-confirmed AD or any AD treatment*** |  |  |  |
| 18 | 1.If any physician had diagnosed eczema during the last 4 years  2.If the child was treated for eczema in the last 12 months" | Yes to 1 or 2 | No to 1 and 2 | (21) |
|  | ***Physician-confirmed AD or parent-reported chronic and recurrent itchy skin condition or medical record*** |  |  |  |
| 19 | 1. Has your child had itchy rash? 2. Has a doctor told you that your child had atopic dermatitis? 3. Was the rash recurrent? 4. In which 0.5 month periods did your child have the rash? 5. Where was the rash located?  6. Hospital discharge recorded of ICD 10 code L20 | 1. Yes  2. Yes  3. Yes  4. Rash for at least 4 consecutive 0.5-month periods | No AD or AD symptoms recorded in interview, questionnaire, or registered data | (1, 22) |
|  |  | 5. localization in elbow creases,  behind the knees, face, wrists/hands or generalized/ 4 or more localizations  Yes to 1 or 2 and Yes to 3 or 4 and 5 or 6 | |  |
|  | ***Physician-confirmed AD, parent-reported chronic itchy skin condition affecting skin creases or physical examination*** |  |  |  |
| 20 | 1. a reported diagnosis by the family physician or paediatrician  2. Parental reporting of dry skin and at least 3 of 4 relevant symptoms (cheek eczema, eczema at other sites, infra auricular fissuring, and scaly or itchy rash for a longer time period)"  3. Visible AD at skin examination as defined above. | Yes to 1, 2 or 3 | No to 1, 2, and 3 | (23) |

**AD definitions for cumulative prevalence based on parent-reported AD**

| No. | Definition of eczema | Case response | Control response | Ref |
| --- | --- | --- | --- | --- |
|  | ***Parent-reported AD*** |  |  |  |
| 21 | 1. Has your child ever had atopic dermatitis? | Yes to 1 | No to 1 | (1, 24) |
| 22 | 1. Has your child ever suffered from eczema? | Yes to 1 | No to 1 | (1, 25) |
| 23 | 1. Has your child ever experienced atopic eczema since birth to six months, and from 7 to 14 months’? | Yes to 1 | No to 1 | (26) |
| 24 | 1. Whether their child had had any eczematous rash during the first year of life or at any time after the first year. | Yes to 1 | No to 1 | (27) |
|  | ***Parent-reported itchy chronic skin condition affecting skin creases*** |  |  |  |
| 25 | 1. chronic or chronically relapsing, itchy dermatitis (lasting more than 6 weeks) with characteristic morphology (areas of scaly, erythematous, pruritic lesions) and distribution (face, post-auricular area, scalp, extensor surface or extremities and flexural creases) | Yes to 1 | No to 1 | (28) |
| 26 | 1. Schultz Larsen criteria | Fulfilling Schultz Larsen criteria | Not fulfilling Schultz Larsen criteria | (29) |
|  | ***Parent-reported itchy skin condition affecting skin creases*** |  |  |  |
| 27 | 1. Has your child ever had an itchy skin rash which has affected the skin creases, e.g., the folds of the elbow or behind the knees? | Yes to 1 | No to 1 | (30) |
|  | ***Parent-reported AD affecting skin creases*** |  |  |  |
| 28 | 1. "Did he/she have infantile (baby) eczema?"  2. "Has he/she ever had eczema in the creases of the elbows or wrists of knees?" | Yes to 1 or 2 | No to 1 and 2 | (1, 31) |
|  | ***Parent-reported AD or use of medication*** |  |  |  |
| 29 | 1. Has your child ever suffered from atopic eczema  2. Has your child suffered from atopic eczema during the last 12 months?  3. Has your child used eczema medication during the last 12 months? | Yes to 1, 2 or 3 | No to 1, 2, and 3 | (3) |
|  | ***Parent-reported AD, parent-reported chronic itchy skin condition, or medical record*** |  |  |  |
| 30 | 1. Has your child ever had atopic dermatitis?  2. Has your child ever had an itchy rash which was coming and going for at least 6 months? 3. Hospital discharge record of ICD10 code L20 | Yes to [1 and 2] or 3 | No to [1 or 2] and 3 | (1, 22) |
|  | ***Parent-reported AD or physical examination*** |  |  |  |
| 31 | 1. History of atopic dermatitis in first year?  2. History of atopic dermatitis after first year?  3. Medical examination | Yes to 1, 2, or 3 | No to 1, 2, and 3 | (1, 27) |
|  | ***Parent-reported AD affecting skin creases or physical examination*** |  |  |  |
| 32 | 1. parent reporting of eczema and flexural involvement  2. visible atopic dermatitis at the time of physical examination" | Yes to 1 or 2 | No to 1 and 2 | (23) |

**AD definitions for annual prevalence based on physician-confirmed AD**

| No. | Definition of eczema in previous papers | Case response | Control response | Ref |
| --- | --- | --- | --- | --- |
|  | ***Physician-confirmed AD*** |  |  |  |
| 33 | 1. Has your child ever had atopic dermatitis?  2. Did a doctor ever diagnose atopic dermatitis in your child?  3. Did your child have atopic dermatitis during the past 12 months? | Yes to 1, 2, and 3 | No to 1, 2, and 3 | (1, 2) |
| 34 | 1. Was your child diagnosed with eczema in the last 6 months/last year? (Not as far as I know; yes, did not go to the doctor; yes, went to the general practitioner(GP); yes, went to the hospital) | Yes, went to GP or yes, went to the hospital | No to 1 or Yes, did not got to Dr | (32) |
| 35 | 1. Did a physician diagnose your child having atopic dermatitis in the past 6/12 months? | Yes to 1 | No to 1 | (1, 33) |
| 36 | 1. Has your child had atopic dermatitis in the last 12 months? saw a doctor, did not saw a doctor, no | Yes, saw a doctor | No or did not saw a Dr | (1, 24) |
| 37 | 1. Has your baby had eczema? (doctor diagnosed eczema) | Yes to 1 | No to 1 | (34) |
|  | ***Physician-confirmed AD and parent-reported itchy skin condition*** |  |  |  |
| 38 | 1. current itchy rash (occurring in the last 12 months)  2. given a diagnosis of eczema at some stage in their life | Yes to 1 and 2 | No to 1 or 2 | (35) |
|  | ***Physician-confirmed AD and parent-reported chronic itchy skin condition affecting skin creases*** |  |  |  |
| 39 | 1. an itchy rash that was coming and going during the last 12 months (at folds of the elbows, or back of the knee, or front of the ankles, or in the neck, or around eyes and ears)"  2. a doctor’s diagnosis of eczema ever. | Yes to 1 and 2 | No to 1 or 2 | (36) |
|  | ***Physician-confirmed AD and parent-reported chronic skin condition affecting skin creases*** |  |  |  |
| 40 | 1. a diagnosis of AD by a physician  2. the indication of characteristic skin alterations in the typical predilection skin areas (face, neck, and flexural folds of the extremities) for more than 6 months during the 12 months before the follow ups | Yes to 1 and 2 | No to 1 or 2 | (37) |
|  | ***Physician-confirmed AD or use of anti-inflammatory medication and parent-reported chronic itchy skin condition*** | |  |  |
| 41 | 1. those reporting itchy rashes with age-specific location for 2 weeks or more  2. use of topical corticosteroids in the past 12 months  3. doctor’s diagnosis of eczema in the past 24 months | Yes to [1 and 3] or [2 and 3] | No to any two of 1, 2, and 3 | (4) |
|  | ***Physician-confirmed AD and use of anti-inflammatory medication*** |  |  |  |
| 42 | 1. Has your child been diagnosed with eczema by a physician after the age of one year?  2. Has your child been treated with cortisone ointment for eczema during the last 12 months?" | Yes to 1 and 2 | No to 1 or 2 | (34) |
|  | ***Physician-confirmed AD or physical examination*** |  |  |  |
| 43 | 1. Has a doctor diagnosed your child with allergic or atopic eczema in the past 6 months?  2. clinical examination using UK diagnostic criteria for AD | Yes to 1 or presence of AD | No to 1 and 2 | (21) |
|  | ***Physician-confirmed AD and parent-reported AD or use of anti-inflammatory medication and other condition*** |  |  |  |
| 44 | 1. Has your child been diagnosed with eczema by a physician?  2. Has your child had symptoms of eczema during the last 12 months  3. Has your child been treated with cortisone ointment for eczema during the last 12 months?  4. At what age did your child first show symptoms of eczema? | 1. Yes  2. Yes  3. Yes  4. Unknown  Yes to 1, [2 or 3] , and 4 | No to 1 or No to 2 and 3 | (34) |

**AD definitions for annual prevalence based on both of physician-confirmed and parent-reported AD**

| No. | Operational definition of eczema in previous papers | Case response | Control response | Ref |
| --- | --- | --- | --- | --- |
|  | ***Physician-confirmed AD or parent-reported itchy skin condition affecting skin creases*** |  |  |  |
| 45 | 1. Dry skin, itchy rashes with age-specific location for 2 weeks or more  2. Doctor’s diagnosis of eczema in the past 12 months. | Yes to 1 or 2 | No to 1 and 2 | (4) |
| 46 | 1. Dry skin in combination with itchy rash for at least 2 weeks, with typical localization, during the last 12 months  2. Doctor's diagnosis of eczema during the last 24 months | Yes to 1 or 2 | No to 1 and 2 | (38) |

**AD definitions for annual prevalence based on parent-reported AD**

| No. | Definition of eczema | Case response | Control response | Ref |
| --- | --- | --- | --- | --- |
|  | ***Parent-reported AD*** |  |  |  |
| 47 | 1. self- reported eczema in the last 12 months | Yes to 1 | No to 1 | (27) |
|  | ***Parent-reported chronic itchy skin condition*** |  |  |  |
| 48 | 1. Has your child ever had an itchy rash which was coming and going for at least 6 months?  2. Has your child had this itchy rash in the last 12 months?" | Yes to 1 and 2 | No to 1 or 2 | (39) |
| 49 | 1. Has your child had an itchy rash coming and going for the period of at least 6 months?  2. Has your child had this itchy rash in the last 12 months?" | Yes to 1 and 2 | No to 1 or 2 | (40) |
| 50 | 1. ever eczema  2. itchy rash during the previous 12 months" | Yes to 1 and 2 | No to 1 or 2 | (41) |
|  | ***Parent-reported chronic skin condition*** |  |  |  |
| 51 | 1. Has the baby had rashes on his/her body that look like the rash in these picture in the past 6 months?  2. Was the rash recurrent? | Yes to 1 and 2 | No to 1 or 2 | (42) |
|  | ***Parent-reported chronic itchy skin condition affecting skin creases*** |  |  |  |
| 52 | 1. an itchy rash that persisted for at least 6 months and was located in the antecubital or popliteal fossae, wrists, ankles, neck or face during the last 12 months. | Yes to 1 | No to 1 | (43) |
|  | ***Parent-reported itchy skin condition affecting skin creases*** |  |  |  |
| 53 | 1. Has the baby had a rash in the joints and creases of her body (e.g. behind the knees, under the arms)?  2. Has she had an itchy, dry, oozing or crusted rash on the face, forearms or shins?" | Yes to 1 or 2 | No to 1 and 2 | (44) |
| 54 | 1. Has she had a skin rash in the joints and creases of her body (e.g. behind the knees, elbows, under the arms) since she was 6 months old?  2. Has she had an itchy, dry, oozing or crusted rash on the face, forearms or shins since she was 6 months old? | Yes to 1 or 2 | No to 1 and 2 | (44) |
| 55 | 1. Has your child has an itchy, dry skin rash in the joints and creases of her body (e.g. behind the knees, elbows, under the arms) in the past 12 months | Yes to 1 | No to 1 | (44) |
|  | ***Parent-reported AD or use of AD medication*** |  |  |  |
| 56 | 1. Has your child suffered from atopic eczema during the last 12 months?  2. Has your child used eczema medication during the last 12 months? | Yes to 1 or 2 | No to 1 and 2 | (3) |
|  | ***Parent-reported skin condition or physical examination*** |  |  |  |
| 57 | 1. Whether their child had had eczematous rashes in the past 12months?  2. visible eczema recorded by experienced school medical officers | Yes to 1 or presence of eczema to 2 | No to 1 and 2 | (27) |
|  | ***Parent-reported chronic itchy skin condition affecting skin creases and other condition or physical examination*** |  |  |  |
| 58 | 1. the child to have had an itchy skin condition during the previous 12 months plus at least 3 or more of the following: (1) involvement of skin creases and cheeks; (2) history of allergic disease in siblings or parents; (3) history of generally dry skin; and (4) visible flexural dermatitis, including that affecting the cheeks, forehead and outer limbs | Fulfilling UK working party criteria |  | (45, 46) |

**AD definitions for unclear period of prevalence**

| No. | Definition of eczema | Case response | Control response | Ref |
| --- | --- | --- | --- | --- |
|  | ***Physician-confirmed AD and chronic skin condition affecting skin creases*** |  |  |  |
| 59 | 1. physician-diagnosed allergy diseases, including atopic dermatitis (chronic or relapsing dermatitis with erythematous, scaly or itchy rashes on the face, neck, anterior chest wall, extensor areas or the flexural folds of extremities) | Yes to 1 | No to 1 | (47) |

**Table E2.**  Transition between case and control in each definition

Number and percentage of those children defined as Case by each definition assigned as Controls in other definitions.

|  |  | Number (%) of children assigned as controls in other definitions | | | | |
| --- | --- | --- | --- | --- | --- | --- |
| MAAS |  | Definition 1 | | Definition 2 | Definition 3 | Definition 4 |
| Cases by: | Definition 1  (n = 263) | - | | 32 (12) | 32 (12) | 0 (0) |
|  | Definition 2  (n = 233) | 2 (0.9) | | - | 0 (0) | 0 (0) |
|  | Definition 3  (n = 289) | 58 (20) | | 56 (19) | - | 0 (0) |
|  | Definition 4  (n = 324) | 58 (18) | | 88 (27) | 32 (10) | - |
| Ashford |  | Definition 1 | Definition 2 | | Definition 3 | Definition 4 |
| Cases by: | Definition 1  (n = 114) | - | 41 (36) | | 41 (36) | 0 (0) |
|  | Definition 2  (n = 73) | 0 (0) | - | | 0 (0) | 0 (0) |
|  | Definition 3  (n = 89) | 16 (18) | 16 (18) | | - | 0 (0) |
|  | Definition 4  (n = 130) | 16 (12) | 57 (43) | | 41 (32) | - |

**Table E3.** Associates of AD using 4 different “Case” definitions.

Definition 1: Physician-confirmed AD; Definition 2: Physician-confirmed AD and parent-reported chronic itchy skin condition affecting skin creases; Definition 3: Parent-reported chronic itchy skin condition affecting skin creases; and Definition 4: Physician-confirmed AD or parent-reported chronic itchy skin condition affecting skin creases.

ORs: odds ratios; C.I.: confident interval; Binary logistic regression

|  | Parental eczema |  |  | Allergic  sensitization |  |  | Asthma |  |
| --- | --- | --- | --- | --- | --- | --- | --- | --- |
|  | ORs  (95% C.I.) | P value |  | ORs  (95% C.I.) | P value |  | ORs  (95% C.I.) | P value |
| MAAS |  |  |  |  |  |  |  |  |
| Definition 1 | 1.8  (1.3 - 2.4) | <.001 |  | 2.9  (2.2 - 4.0) | <.001 |  | 2.2  (1.6 - 3.0) | <.001 |
| Definition 2 | 1.7  (1.3 - 2.4) | .001 |  | 3.4  (2.5 - 4.7) | <.001 |  | 2.2  (1.6 - 3.0) | <.001 |
| Definition 3 | 1.6  (1.2 - 2.1) | .003 |  | 3.4  (2.5 - 4.5) | <.001 |  | 1.8  (1.4 - 2.5) | <.001 |
| Definition 4 | 1.6  (1.2 - 2.1) | .002 |  | 3.0  (2.3 - 4.1) | <.001 |  | 1.9  (1.4 - 2.6) | <.001 |
| Ashford |  |  |  |  |  |  |  |  |
| Definition 1 | 1.3  (0.8 - 2.0) | .27 |  | 3.6  (2.2 - 6.1) | <.001 |  | 2.3  (1.4 - 3.6) | <.001 |
| Definition 2 | 1.3  (0.8 - 2.3) | .29 |  | 5.4  (3.1 - 9.6) | <.001 |  | 2.5  (1.4 - 4.2) | .001 |
| Definition 3 | 1.4  (0.8 - 2.2) | .19 |  | 4.5  (2.6 - 7.8) | <.001 |  | 2.0  (1.2 - 3.4) | .006 |
| Definition 4 | 1.3  (0.9 - 2.0) | .18 |  | 3.3  (2.0 - 5.6) | <.001 |  | 2.0  (1.3 - 3.2) | .002 |

**Table E4.** AD definitions for “Controls”

Question 1 (physician-confirmed ever AD) “Has a doctor ever told that your child had eczema?” and “Has a doctor ever told your son or daughter has eczema?”;

Question 2 (current itchy skin condition) ”Has your child had an itchy rash at any time in the last 12 months” and “In the last twelve months, has your son had an itchy skin rash? (by itchy we mean scratching or rubbing the skin)”;

Question 3 (current flexural rash) “Has this itchy rash at any time affected any of the following places: the fold of the elbows, behind the knees; in front of the ankles, under the buttocks; around the neck, ear or eyes?” and “Has this skin condition at any time affected the skin creases in the past? (by skin creases we mean fronts of elbows, behind the knees, fronts of ankles”.

| Version | Control response | Case response |
| --- | --- | --- |
| Strict | Negatives to all 3 questions | Yes to question 1 and 2 or 2 and 3 |
| Moderate | Negatives to at least 2 questions | Same as above |

**Table E5.** Questions with a positive response among unclassifiable children in the “strict” definition for “controls”.

Question 1 (physician-confirmed ever AD) “Has a doctor ever told you that your child had eczema?” and “Has a doctor ever told you that your son or daughter has eczema?”;

Question 2 (current itchy skin condition) ”Has your child had an itchy rash at any time in the last 12 months” and “In the last twelve months, has your son had an itchy skin rash? (by itchy we mean scratching or rubbing the skin)”;

Question 3 (current flexural rash) “Has this itchy rash at any time affected any of the following places: the fold of the elbows, behind the knees; in front of the ankles, under the buttocks; around the neck, ear or eyes?” and “Has this skin condition at any time affected the skin creases in the past? (by skin creases we mean fronts of elbows, behind the knees, fronts of ankles”

|  | **MAAS (N=186)** | **Ashford (N=135)** |
| --- | --- | --- |
|  | n (%) | n (%) |
| Question 1: Physician-confirmed ever AD | 156 (84%) | 100 (74%) |
| Question 2: Current itchy skin condition | 22 (12%) | 35 (26%) |
| Question 3: Current flexural rash | 0 (0%) | 0 (0%) |

In addition eight children in MAAS were assigned to “Unclassifiable” due to the missing value of physician-confirmed ever AD. None of the children with a positive answer to question 3 was assigned to “Unclassifiable” because only children who gave a positive answer to question 2 answered question 3.

**Table E6.** Association of children defined as “unclassifiable” in “strict” control definition with *Filaggrin* null mutations**.**

Children with only two negatives to three questions in the “strict” control version were assigned in unclassifiable group. Odds ratios for FLG mutations were obtained from a binary logistic analysis. The reference is children assigned to the control.

| Control definition | **MAAS (N = 1069)** | |  |  | **Ashford (N = 604)** | |  |
| --- | --- | --- | --- | --- | --- | --- | --- |
|  | N (%) | ORs (95% CI) | P value |  | N (%) | ORs (95% CI) | P value |
| Unclassifiable | 186 (18) | 2.5 (1.3 – 4.6) | 0.005 |  | 135 (22) | 1.4 (0.6 - 3.1) | 0.387 |

**Table E7.** Odds ratios for the association between AD and different risk factors in two different “Control” definitions using same case definition (Definition 4: Physician-confirmed AD or parent-reported chronic itchy skin condition affecting skin creases).

ORs: odds ratios; 95% C.I.: 95% confident interval; Binary logistic regression

|  | Parental eczema |  |  | Allergic  sensitization |  |  | Asthma |  |
| --- | --- | --- | --- | --- | --- | --- | --- | --- |
|  | ORs  (95% C.I.) | P value |  | ORs  (95% C.I.) | P value |  | ORs  (95% C.I.) | P value |
| MAAS |  |  |  |  |  |  |  |  |
| Strict | 1.7  (1.2 - 2.3) | .001 |  | 3.7  (2.7 - 5.1) | <.001 |  | 2.4  (1.7 - 3.3) | <.001 |
| Moderate | 1.6  (1.2 - 2.1) | .002 |  | 3.0  (2.3 - 4.1) | <.001 |  | 1.9  (1.4 - 2.6) | <.001 |
|  |  |  |  |  |  |  |  |  |
| Ashford |  |  |  |  |  |  |  |  |
| Strict | 1.2  (0.8 - 1.9) | .36 |  | 4.6  (2.6 – 8.2) | <.001 |  | 2.3  (1.4 – 3.8) | .001 |
| Moderate | 1.3  (0.9 - 2.0) | .19 |  | 3.3  (2.0 - 5.6) | <.001 |  | 2.0  (1.3 - 3.2) | .002 |

**REFERENCES**

1. Paternoster L, Standl M, Chen C-m, Ramasamy A, Bønnelykke K, Duijts L, et al. Meta-analysis of genome-wide association studies identifies three new risk loci for atopic dermatitis. Nature genetics. 2011;44(2):187-92.

2. Brunekreef B, Smit J, de Jongste J, Neijens H, Gerritsen J, Postma D, et al. The prevention and incidence of asthma and mite allergy (PIAMA) birth cohort study: design and first results. Pediatr Allergy Immunol. 2002;13 Suppl 15:55-60.

3. Font-Ribera L, Gracia-Lavedan E, Esplugues A, Ballester F, Jiménez Zabala A, Santa Marina L, et al. Water hardness and eczema at 1 and 4 y of age in the INMA birth cohort. Environmental research. 2015;142:579-85.

4. Ballardini N, Kull I, Lind T, Hallner E, Almqvist C, Östblom E, et al. Development and comorbidity of eczema, asthma and rhinitis to age 12 - Data from the BAMSE birth cohort. Allergy: European Journal of Allergy and Clinical Immunology. 2012;67(4):537-44.

5. Bisgaard H, Simpson A, Palmer CNA, Bønnelykke K, McLean I, Mukhopadhyay S, et al. Gene-Environment Interaction in the Onset of Eczema in Infancy: Filaggrin Loss-of-Function Mutations Enhanced by Neonatal Cat Exposure. PLoS Medicine. 2008;5(6):e131-e.

6. Kim JP, Chao LX, Simpson EL, Silverberg JI. Persistence of atopic dermatitis (AD): A systematic review and meta-analysis. Journal of the American Academy of Dermatology. 2016;75(4):681-7.e11.

7. Ferreira MA, Matheson MC, Duffy DL, Marks GB, Hui J, Le Souëf P, et al. Identification of IL6R and chromosome 11q13.5 as risk loci for asthma. Lancet. 2011;378(9795):1006-14.

8. Magnus P, Irgens LM, Haug K, Nystad W, Skjaerven R, Stoltenberg C, et al. Cohort profile: the Norwegian Mother and Child Cohort Study (MoBa). Int J Epidemiol. 2006;35(5):1146-50.

9. Rantakallio P. The longitudinal study of the northern Finland birth cohort of 1966. Paediatr Perinat Epidemiol. 1988;2(1):59-88.

10. Schmitt J, Apfelbacher C, Chen C-M, Romanos M, Sausenthaler S, Koletzko S, et al. Infant-onset eczema in relation to mental health problems at age 10 years: Results from a prospective birth cohort study (German Infant Nutrition Intervention plus). Journal of Allergy and Clinical Immunology. 2010;125(2):404-10.

11. Williams LA, Evans SF, Newnham JP. Prospective cohort study of factors influencing the relative weights of the placenta and the newborn infant. BMJ. 1997;314(7098):1864-8.

12. Golding J, Pembrey M, Jones R, Team AS. ALSPAC--the Avon Longitudinal Study of Parents and Children. I. Study methodology. Paediatr Perinat Epidemiol. 2001;15(1):74-87.

13. Kull I, Melen E, Alm J, Hallberg J, Svartengren M, van Hage M, et al. Breast-feeding in relation to asthma, lung function, and sensitization in young schoolchildren. J Allergy Clin Immunol. 2010;125(5):1013-9.

14. Boomsma DI, de Geus EJ, Vink JM, Stubbe JH, Distel MA, Hottenga JJ, et al. Netherlands Twin Register: from twins to twin families. Twin Res Hum Genet. 2006;9(6):849-57.

15. Beck C, Koplin J, Dharmage S, Wake M, Gurrin L, McWilliam V, et al. Persistent Food Allergy and Food Allergy Coexistent with Eczema Is Associated with Reduced Growth in the First 4 Years of Life. The Journal of Allergy and Clinical Immunology: In Practice. 2016;4(2):248-56.e3.

16. Hartwig IRV, Sly PD, Schmidt LA, Van Lieshout RJ, Bienenstock J, Holt PG, et al. Prenatal adverse life events increase the risk for atopic diseases in children, which is enhanced in the absence of a maternal atopic predisposition. Journal of Allergy and Clinical Immunology. 2014;134(1):160-9.e7.

17. Papathoma E, Triga M, Fouzas S, Dimitriou G. Cesarean section delivery and development of food allergy and atopic dermatitis in early childhood. Pediatric Allergy and Immunology. 2016;27(4):419-24.

18. Böhme M, Söderhäll C, Kull I, Bergström A, van Hage M, Wahlgren C-F. Filaggrin mutations increase the risk for persistent dry skin and eczema independent of sensitization. The Journal of allergy and clinical immunology. 2012;129(4):1153-5.

19. Grönhagen C, Lidén C, Wahlgren CF, Ballardini N, Bergström A, Kull I, et al. Hand eczema and atopic dermatitis in adolescents: a prospective cohort study from the BAMSE project. British Journal of Dermatology. 2015;173(5):1175-82.

20. Ballardini N, Bergström A, Wahlgren CF, van Hage M, Hallner E, Kull I, et al. IgE antibodies in relation to prevalence and multimorbidity of eczema, asthma, and rhinitis from birth to adolescence. Allergy. 2016;71(3):342-9.

21. Filipiak-Pittroff B, Schnopp C, Berdel D, Naumann A, Sedlmeier S, Onken A, et al. Predictive value of food sensitization and filaggrin mutations in children with eczema. Journal of Allergy and Clinical Immunology. 2011;128(6).

22. Harpsøe MC, Basit S, Bager P, Wohlfahrt J, Benn CS, Nøhr EA, et al. Maternal obesity, gestational weight gain, and risk of asthma and atopic disease in offspring: A study within the Danish National Birth Cohort. Journal of Allergy and Clinical Immunology. 2013;131(4):1033-40.

23. Illi S, von Mutius E, Lau S, Nickel R, Grüber C, Niggemann B, et al. The natural course of atopic dermatitis from birth to age 7 years and the association with asthma. The Journal of allergy and clinical immunology. 2004;113(5):925-31.

24. Jaddoe VW, van Duijn CM, van der Heijden AJ, Mackenbach JP, Moll HA, Steegers EA, et al. The Generation R Study: design and cohort update 2010. Eur J Epidemiol. 2010;25(11):823-41.

25. Custovic A, Simpson BM, Murray CS, Lowe L, Woodcock A. The National Asthma Campaign Manchester Asthma and Allergy Study. Pediatric allergy and immunology : official publication of the European Society of Pediatric Allergy and Immunology. 2002;13 Suppl 1:32-7.

26. Morales E, García-Esteban R, Guxens M, Guerra S, Mendez M, Moltó-Puigmartí C, et al. Effects of prolonged breastfeeding and colostrum fatty acids on allergic manifestations and infections in infancy. Clinical and experimental allergy : journal of the British Society for Allergy and Clinical Immunology. 2012;42(6):918-28.

27. Williams, Strachan. The natural history of childhood eczema: observations from the British 1958 birth cohort study. British Journal of Dermatology. 1998;139(5):834-9.

28. Tariq SM, Matthews SM, Hakim EA, Stevens M, Arshad SH, Hide DW. The prevalence of and risk factors for atopy in early childhood: a whole population birth cohort study. The Journal of allergy and clinical immunology. 1998;101(5):587-93.

29. Mortz CG, Andersen KE, Dellgren C, Barington T, Bindslev-Jensen C. Atopic dermatitis from adolescence to adulthood in the TOACS cohort: Prevalence, persistence and comorbidities. Allergy. 2015;70(7):836-45.

30. Amberbir A, Medhin G, Alem A, Britton J, Davey G, Venn A. The Role of Acetaminophen and Geohelminth Infection on the Incidence of Wheeze and Eczema. American Journal of Respiratory and Critical Care Medicine. 2011;183(2):165-70.

31. Martin PE, Matheson MC, Gurrin L, Burgess JA, Osborne N, Lowe AJ, et al. Childhood eczema and rhinitis predict atopic but not nonatopic adult asthma: A prospective cohort study over 4 decades. Journal of Allergy and Clinical Immunology. 2011;127(6):1473-9.e1.

32. Gazibara T, Elbert NJ, den Dekker HT, de Jongste JC, Reiss I, McGrath JJ, et al. Associations of maternal and fetal 25-hydroxyvitamin D levels with childhood eczema: The Generation R Study. Pediatric Allergy and Immunology. 2016;27(3):283-9.

33. Heinrich J, Bolte G, Hölscher B, Douwes J, Lehmann I, Fahlbusch B, et al. Allergens and endotoxin on mothers' mattresses and total immunoglobulin E in cord blood of neonates. Eur Respir J. 2002;20(3):617-23.

34. Goksör E, Loid P, Alm B, Åberg N, Wennergren G. The allergic march comprises the coexistence of related patterns of allergic disease not just the progressive development of one disease. Acta paediatrica (Oslo, Norway : 1992). 2016;105(12):1472-9.

35. Kurukulaaratchy R, Fenn M, Matthews S, Hasan Arshad S. The prevalence, characteristics of and early life risk factors for eczema in 10-year-old children. Pediatric Allergy and Immunology. 2003;14(3):178-83.

36. van Elten TM, van Rossem L, Wijga AH, Brunekreef B, de Jongste JC, Koppelman GH, et al. Breast milk fatty acid composition has a long-term effect on the risk of asthma, eczema, and sensitization. Allergy. 2015;70(11):1468-76.

37. Peters AS, Kellberger J, Vogelberg C, Dressel H, Windstetter D, Weinmayr G, et al. Prediction of the incidence, recurrence, and persistence of atopic dermatitis in adolescence: A prospective cohort study. Journal of Allergy and Clinical Immunology. 2010;126(3):590-5.e3.

38. Kull I, Bergstrom A, Lilja G, Pershagen G, Wickman M. Fish consumption during the first year of life and development of allergic diseases during childhood. Allergy. 2006;61(8):1009-15.

39. von Kobyletzki LB, Bornehag C-G, Hasselgren M, Larsson M, Lindström CB, Svensson Å. Eczema in early childhood is strongly associated with the development of asthma and rhinitis in a prospective cohort. BMC dermatology. 2012;12:11-.

40. Semic-Jusufagic A, Gevaert P, Bachert C, Murray C, Simpson A, Custovic A. Increased serum-soluble interleukin-5 receptor alpha level precedes the development of eczema in children. Pediatric Allergy and Immunology. 2010;21(7):1052-8.

41. Ziyab AH, Raza A, Karmaus W, Tongue N, Zhang H, Matthews S, et al. Trends in eczema in the first 18 years of life: results from the Isle of Wight 1989 birth cohort study. Clinical & Experimental Allergy. 2010;40(12):1776-84.

42. Sangsupawanich P, Chongsuvivatwong V, Mo-Suwan L, Choprapawon C. Relationship between atopic dermatitis and wheeze in the first year of life: analysis of a prospective cohort of Thai children. Journal of investigational allergology & clinical immunology. 2007;17(5):292-6.

43. Gough H, Grabenhenrich L, Reich A, Eckers N, Nitsche O, Schramm D, et al. Allergic multimorbidity of asthma, rhinitis and eczema over 20 years in the German birth cohort MAS. Pediatric Allergy and Immunology. 2015;26(5):431-7.

44. Perkin M, Strachan DP, Williams HC, Kennedy CTC, Golding J, The AST. Natural history of atopic dermatitis and its relationship to serum total immunoglobulin E in a population -based birth cohort study. Pediatric Allergy and Immunology. 2004;15(3):221-9.

45. Cooper PJ, Chico ME, Amorim LD, Sandoval C, Vaca M, Strina A, et al. Effects of maternal geohelminth infections on allergy in early childhood. The Journal of allergy and clinical immunology. 2016;137(3):899-906.e2.

46. Purvis DJ, Thompson JMD, Clark PM, Robinson E, Black PN, Wild CJ, et al. Risk factors for atopic dermatitis in New Zealand children at 3.5 year of age. British Journal of Dermatology. 2005;152(4):742-9.

47. Lee M-T, Wu C-C, Ou C-Y, Chang J-C, Liu C-A, Wang C-L, et al. A prospective birth cohort study of different risk factors for development of allergic diseases in offspring of non-atopic parents. Oncotarget. 2017;8(7):10858-70.
